# Fine Detection of Human Motion During Activities of Daily Living as a Clinical Indicator for the Detection and Early Treatment of Chronic Diseases: The E-Mob Project

**DOI:** 10.2196/32362

**Published:** 2022-01-14

**Authors:** David Thivel, Alice Corteval, Jean-Marie Favreau, Emmanuel Bergeret, Ludovic Samalin, Frédéric Costes, Farouk Toumani, Christian Dualé, Bruno Pereira, Alain Eschalier, Nicole Fearnbach, Martine Duclos, Anne Tournadre

**Affiliations:** 1 Clermont Auvergne University Aubiere France; 2 Analgesia Foundation Clermont-Ferrand France; 3 Clermont Ferrand University Hospital Clermont-Ferrand France; 4 Pennington Biomedical Research Center Baton Rouge, LA United States

**Keywords:** indicator, fine body motion, movement behaviors, decomposition, structuration, sequencing

## Abstract

Methods to measure physical activity and sedentary behaviors typically quantify the amount of time devoted to these activities. Among patients with chronic diseases, these methods can provide interesting behavioral information, but generally do not capture detailed body motion and fine movement behaviors. Fine detection of motion may provide additional information about functional decline that is of clinical interest in chronic diseases. This perspective paper highlights the need for more developed and sophisticated tools to better identify and track the decomposition, structuration, and sequencing of the daily movements of humans. The primary goal is to provide a reliable and useful clinical diagnostic and predictive indicator of the stage and evolution of chronic diseases, in order to prevent related comorbidities and complications among patients.

## Introduction

The alarming increases in physical inactivity and sedentary behaviors that accompanied societal development have favored the progression of chronic diseases. More patients now require ongoing medical attention and have limitations on activities of daily living, leading to reductions in life expectancy and health span [1]. Public health recommendations today advocate for the adoption of a minimal amount of moderate-to-vigorous physical activity (MVPA) each day, with a limited time devoted to sedentary activities (eg, daily sitting and screen time) [2]. While scientific societies and public health agencies long focused on the promotion of physical activity (PA), they also now include sedentary guidelines for overall health, with sedentary time independent of an individual’s PA level [2]. According to Ekelund et al, 65 minutes per day of MVPA may be required to counteract the negative effects of 6 to 7 hours of daily sitting time on overall health [3]. These results clearly highlight the need for the joint promotion of both PA and reduced sedentary time. While these epidemiological results and associated recommendations are based on the general population, data regarding the relationship between the PA/sedentary profile of patients with chronic diseases and the bidirectional associations with disease progression and comorbidity are insufficient.

Methods to measure PA and sedentary behaviors typically quantify the amount of time devoted to these activities, estimating their intensity and frequency. Estimates of energetic costs (in metabolic equivalents of task [METs]) and energy expenditure are also available from most methods. The large majority of tools and technologies (eg, interviews, questionnaires, and wearable devices) that are available for tracking PA/sedentary behavior use these metrics. There are numerous reports of the validity and reliability/reproducibility of these tools among patients with various diseases, but the results are relatively poor to modest [4-6]. Well-conducted reviews (with systematic and meta-analytic approaches) have discussed in detail the inherent methodological limitations of the tools and devices commonly used to assess PA and sedentary behavior [7-9].

Moreover, quantifying PA and sedentary behavior among patients with chronic diseases can provide interesting behavioral information, but generally does not capture detailed and fine motions. Fine detection of motion may provide additional information about functional decline that is of clinical interest in chronic diseases. Of interest is the identification and tracking of the decomposition, structuration, and sequencing of humans’ daily movements (meaning here the identification of the involved limbs, their respective contribution, and the temporal order of implication), above the simple quantification of PA and sedentary behavior. These metrics could provide a reliable and useful clinical diagnostic and predictive indicator (as an early sign) of the stage and evolution of chronic diseases and related comorbidities and complications. In the longer term, advancement in this direction could potentially influence treatment strategies (posology, timing/chronobiology, and nature of the treatment).

## Body Movements’ Decomposition Above PA and Sedentary Behavior Quantification

A better evaluation or capture of an individual’s singular motion pattern or movement construction, from a musculoskeletal point of view, may allow practitioners to anticipate or track the evolution of some chronic diseases. In their recent work, Chevance et al proposed such an approach, showing that the anticipation and detection of the early signs of individuals’ movement changes, as an indicator of subsequent critical functional gain or loss, need to be considered as “early warning signals for sudden behavioral changes” [10]. Beyond PA and overall human movements themselves, early findings suggest that sudden gain or loss in complex systems could be predicted through early warning signals [11,12], such as slight changes or fluctuations in human motion and movement patterns. On application to movement behaviors, the early anticipation of imminent body motion disruption or early detection of the first signs of fluctuations might represent a potentially reliable signal for delivering “just-in-time” interventions [13]. In that sense, Chevance et al observed that in adults with obesity, fluctuations in walking patterns were associated with the subsequent occurrence of behavioral losses in the following days, clearly demonstrating the need to develop new accessible methods to properly detect such early signals [10]. This need is clearly illustrated by high-quality clinical studies.

### Clinical Evidence

Krieger et al, for instance, clearly noted the need to better identify subprocesses of movement execution in patients with schizophrenia, also highlighting that some neuroleptic treatments have negative side effects such as the slowing of motor execution [14]. The slowing of movement is nonperceptible and not captured with available activity trackers. Obesity has also been shown to affect patients’ body motion and movement patterns, limiting their upper body range of motion during daily activities [15] or patterns of gait through mediolateral adaptations of their gravity center [16]. Once more, while this remains difficult to track in free-living conditions, the evolution of such movement patterns could be of great interest in the clinical care of these patients. In their work, Oubre et al also underlined that movement decomposition captures the core features of ataxia and may be useful for objective, precise, and frequent assessment of ataxia in home and clinic environments [17]. While not exhaustive, these examples clearly point out the urgent need to develop new strategies and tools to better track and catch the fine-grained evolution of patients’ body movements in addition to the quantification of habitual PA and sedentary time.

### Need for New Technologies

Importantly, such a fine-grained clinical exploration of daily motion does not negate the utility and interest for the activity trackers developed to date, but calls for a new and deeper integration and understanding of their signals and sensing capabilities. Commercialized trackers have shown satisfactory acceptability in capturing the daily routine of individuals. We should build on this platform, developing more complex and sophisticated algorithms to better identify and refine human movement patterns [18]. Such a process has been initiated through the development of human activity recognition (HAR) that uses wearable motion sensors, for which a high level of accuracy in predicting activities has been reported [19,20]. These sensors and algorithms have been shown to be valid and reliable among healthy individuals, but lack sensitivity to properly classify human movement in clinical patients, particularly patients presenting motor and gait impairments [9,21]. Moving forward, there is a need to optimize and validate these existing algorithms among patients with chronic diseases [5]. Previously published studies have shown that the validity of existing algorithms to discriminate sedentary behavior from standing and dynamic body behaviors and activities varies and mainly relies on explorations with reduced sample sizes [6,22-29]. While van Dijk-Huisman et al recently proposed an optimized PA classification based on signals from classical accelerometers that reliably classify sedentary and dynamic activities and detect postural transition among hospitalized patients [30], these approaches need to be developed for free-living conditions. Teams of clinicians and engineers should collaborate on technological innovations in measurement tools to fit patient characteristics and treatment plan needs.

## Perspectives and New Technological Challenges

The vast majority of publications and commercial activity tracking solutions rely on the recognition of less than 10 activities (walking, climbing stairs, cycling, etc) [31]. This semantic description does not reflect the fine movements that would be informative in clinical settings. Moreover, they mainly focus on movements that are less typical in day-to-day life (eg, sports activity sessions), and do not capture the whole spectrum of nonexercise activities and finer motions that characterize our contemporary sedentary lifestyle. Understanding the quality (speed and trajectory) of fine movements, as well as the alteration or improvement in movement sequencing or frequency, would be more informative for describing the onset and progression of chronic diseases.

Several challenges are therefore identified and need to be addressed. First, it will be necessary for sensor devices to meet the following requirements: (1) avoid commercial devices or algorithms that do not retain data in the event of very little movement, as these are precisely the phenotypes we are interested in; (2) assume an autonomy of the order of 1 week, to make realistic the use of the device within the framework of a follow-up at home for patients, by ensuring the capture and the treatment of the signals at a frequency of the order of 50 Hz; (3) use sensors at multiple locations on the body in order to significantly capture fine movements related to sedentary behaviors and activities of daily living; and (4) have sensors with representative measurements (such as an accelerometer and gyroscope). The use of very lightweight neural networks, which work on a network personalization approach to customize the training to each patient, seems to be the most promising approach to date.

One of the primary approaches to reduce the energy cost of storing and transmitting data from sensors will be to move the data processing as close as possible to the sensors. In particular, we plan to use tools, such as Tensorflow light, to embed this processing in low-energy sensor devices (much lower than the power of a smartphone, for example). This will involve designing deep neural networks that take into account this distributed computation, where only partial data will come back from each device in order to finish the processing. We also plan to propose unconnected devices, in order to minimize the electronic complexity of the devices, with the transmission taking place over a wired connection when the devices are recharged. The charging and final data collection device could be integrated into a smart home-type environment, which would be a favorable and ecological solution, with little modification to the habits and facilities of the patients who will be equipped.

Such an approach would first need to define and validate a representative taxonomy of activities, from a musculoskeletal point of view, which could be integrated into a reliable and elaborate processing chain. The first approach would rely on a hierarchical taxonomy able to deeply detail the structure, substructure, and finesse of the detected semantics, and to identify and recognize any potential improvement or degradation linked with the evolution of the pathology.

## Discussion: The E-Mob Project

In the above context and as part of the 2020-2025 scientific priorities that include “Human Mobility and Health” (I-site CAP 20-25, third challenge), the University Clermont Auvergne (Clermont-Ferrand, France) gathered an interdisciplinary group of experts (composed of physicians, physiologists, methodologists, biostatisticians, experts in energy metabolism, as well as computer scientists and developers) with the objective to elaborate and conduct a whole research program (the E-Mob project) aiming at (1) improving our technological abilities to precisely and accurately identify fine human body movements that might be relevant and informative when it comes to chronic diseases and (2) determining potential specific “digital movement signatures” that could help predict and follow the evolution of some chronic diseases and serve as a reliable connected support when it comes to treatment strategies.

Briefly, the main idea of the E-Mob project is to propose the integration of the evaluation of detailed and fine human motions as a real clinical indicator. In that sense, we aimed at developing an in-clinic high-resolution human motion setting to perform regular deep and fine evaluation of patient mobility, and to develop an original sensor device and a dedicated algorithmic process to regularly assess body motion while engaged in PA and sedentary behavior in free-living conditions. The results obtained during this free-living evaluation would be directly uploaded from home to the data center to be analyzed and would help the clinical staff (physicians and physiotherapists, as well as nurses who are most of the time the first interlocutor of patients) determine whether the evolution of the activity pattern of the patient is an early warning signal of disease.

The E-Mob project will then develop new personalized HAR algorithms using artificial intelligence and machine learning, which will permit, from accelerometer signals, a qualitative analysis including the executive realization of movement and the identification of a pattern of activity. These algorithms should be personalized and should consider activities of very low intensity, the movement itself (function and performance), its sequence, breaks in sedentary behavior, and specificities to the underlying disease. Ultimately, this project aims to develop a continuous e-strategy to improve our personalized medicine approach. The need for such an approach based on daily human motion as a crucial early clinical indicator, has been emphasized during the COVID-19 pandemic, with clear links between the reduction of healthy movement behaviors and the progression of diseases, particularly metabolic and mental ones.

**Figure 1:**
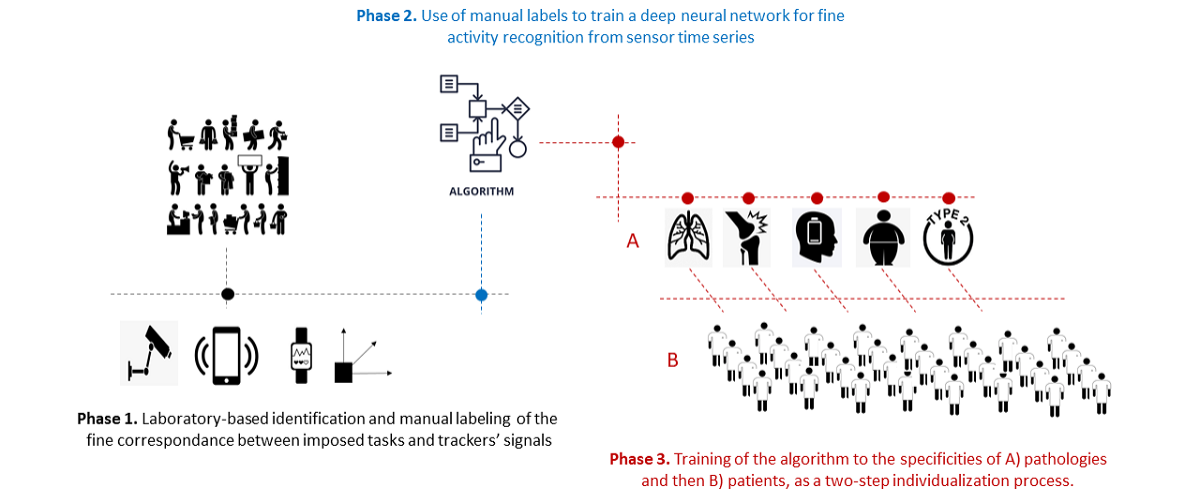
Schematic flow chart of the E-Mob project from Phase 1 (detailed identification of signals from different trackers, specific to predetermined body movements) to Phase 2 (training of the deep neural network) and then Phase 3 (2-step individualization process).

Phase 1 of the project will rely on the identification of specific signals from activity trackers, using a preliminary experiment asking healthy adult individuals to perform preselected and determined movements. This first phase will allow us to isolate the exact signals corresponding to the substages of these movements. From there, new algorithms will be developed in Phase 2. As a 2-step individualization process (Phase 3), these new algorithms will then be tested and trained to the specificities of several chronic diseases and to individual patients in order to identify a digital signature predictive of the state and course of the diseases. We will also capture the development of multimorbidity, focused on the trajectory of each pathology and of each patient. These evaluations will then be replicated during a 4-year longitudinal study. These cohorts will include patients with inflammatory rheumatic diseases (rheumatoid arthritis and spondyloarthritis), and knee and hip osteoarthritis, as well as patients with chronic pain, obesity, type 2 diabetes, chronic obstructive pulmonary disease, major depressive disorders, bipolar disorders, and other mental health conditions. Figure 1 illustrates the steps of the E-Mob project.

## Clinical Implications

There is a clear transition toward the development of more personalized medicine and individualized care, with an essential role for adapted e-technologies. The gross and fine tracking of body motions and the characteristics of these movement patterns over time will have direct clinical implications. The E-Mob solution will allow for the early detection of abnormal movement patterns and fine motions, and allow for targeted care strategies to minimize disease progression. This approach should favor the anticipation of clinical complications and prevent the loss of autonomy or the increased risk of falls associated with chronic diseases. In addition, it may help address the viscous circle of inactivity, isolation, or a hypotonic state in patients with mental and psychological disorders.

This fine tracking of our patients’ daily movements might not only provide information regarding the degradation of their clinical condition but also help evaluate the effect of treatment. Overall, these approaches could aid in decisions regarding clinical care and intervention. Adapted PA programs and therapeutic education have been shown to be appropriate and necessary for most chronic diseases. More finely identifying each patient’s physical limitations, weaknesses, and needs will help practitioners (physiotherapists, adapted educators, and nurses) directly tailor PA prescriptions. Self-monitoring has a positive impact on movement behaviors in both healthy individuals and those with chronic diseases, leading to increased PA and reduced sedentary time, which can translate to long-term beneficial effects.

## Conclusion

Clinical care teams today rely more on e-technologies to develop and improve individualized and personalized medicine. The E-Mob project proposes to integrate such technologies in not only the short-term treatment of patients but also daily life and routine. This approach would allow practitioners to anticipate any evolution of chronic conditions, particularly using premature detection of abnormal fine body motions as an early clinical indicator to consider in care.

## Abbreviations

PA: physical activity
MVPA: moderate-to-vigorous physical activity
HAR: human activity recognition
